# Human Epidermal Growth Factor Receptor 2 (HER2) Expression by Immunohistochemistry and Its Clinical Significance in Hepatocellular Carcinoma: A Single-Center Analysis

**DOI:** 10.7759/cureus.34724

**Published:** 2023-02-07

**Authors:** Denise Magalhães, Joana dos Santos, Amaro Frutuoso, Alexandra Mesquita

**Affiliations:** 1 Oncology, Hospital Pedro Hispano, Matosinhos, PRT; 2 Pathology, Hospital Pedro Hispano, Matosinhos, PRT; 3 Faculty of Medicine, University of Porto, Porto, PRT

**Keywords:** human epidermal growth factor receptor 2, her2, hepatocellular carcinoma, hepatic tumors, molecular analysis, targeted therapy, her2 staining, hepatocellular carcinoma (hcc)

## Abstract

Background: Human epidermal growth factor receptor 2 (HER2) is a member of the tyrosine kinase receptor family. It has been identified as an oncogene and is associated with poor outcomes in multiple tumor types. In hepatocellular carcinoma (HCC), there are contradictory data regarding the HER2 expression and its role in tumor development and progression. Some studies have identified HER2 expression as an early event during tumorigenesis, which decreases with progression and metastasis. Additional data provided evidence that treatment with anti-HER2 therapy resulted in local response and reduction in the metastasis rate in HCC mice models.

Methods: Patients with histological diagnoses of HCC between 2010 and 2020 were included. HER2 staining was performed by immunohistochemistry (IHC), and scoring was done in accordance with the gastric cancer guidelines as 0, 1+, 2+, and 3+. Clinicopathological features were accessed by medical records. This study aims to evaluate HER2 expression by IHC in HCC and to correlate this expression with some clinicopathological features such as Barcelona Clinic Liver Cancer (BCLC) staging, number of hepatic lesions, alpha-fetoprotein level, underlying liver disease, presence of liver cirrhosis, Child-Pugh score, and tumor recurrence.

Results: A total of 57 specimens from 54 patients were included. Of the patients, 85% were men, and the median age at diagnosis was 71 years (interquartile range: 59-75 years). Regarding stage, 61% were at stage 0-A of BCLC. Of the patients, 57% had a solitary HCC nodule. Concerning treatment, surgery was performed in 50% of the patients. HER2 expression was identified in seven patients: five in the membrane and two in the cytoplasm. Concerning the membrane staining, HER2 expression was scored as 1+/2+ in 7.4% (n = 4 patients). Of the patients with HER2 expression, four had a BCLC stage of 0-A and a single HCC nodule; alpha-fetoprotein was <400 ng/mL in all cases. There was no correlation to clinicopathological features. In one patient with HER2 2+ expression at diagnosis, this expression was not identified at tumor progression. Median disease-free survival in HER2 with IHC scores 1+/2+ and cytoplasmatic was 38 months versus 22 months in HER2 with a score of 0 (p = 0.604).

Conclusions: HER2 expression is a rare event in HCC. It was not possible to identify any relation to clinicopathological features. However, when we relate our data to previous trials, HER2 appears to be an early event in the course of HCC.

## Introduction

Primary liver cancer was the sixth most incident and the third most lethal cancer worldwide in 2020. Among all primary liver cancers, hepatocellular carcinoma (HCC) is the most common, accounting for 75-85% of all cases [1].

Hepatic resection is the treatment with curative intent in patients with early-stage HCC (Barcelona Clinic Liver Cancer (BCLC) stage 0-A), good performance status, and preserved liver function. Nevertheless, the recurrence rate at five years is about 50-70%, mainly in the first two years [1-4]. At advanced stages, the benefit of systemic therapy was three months in overall survival (OS): from 7.9 months with placebo to 10.7 months with sorafenib in the SHARP trial [5]. Lenvatinib showed non-inferior results in phase III REFLECT trial [6]. Since then, the first regimen that demonstrated improvement in OS was the combination of anti-programmed death-ligand 1 (anti-PDL1) antibody (atezolizumab) with vascular endothelial growth factor (VEGF) antibody (bevacizumab) in phase III IMbrave150 trial [7,8]. In this trial, the updated analysis showed an OS of 19.2 months in the study arm versus 13.4 months in the standard of care arm (sorafenib; hazard ratio (HR): 0.66) [7,8].

The failure to improve HCC prognosis encouraged research in HCC biology and mutations with the potential of targeted therapies.

Human epidermal growth factor receptor 2 (HER2) is a member of the tyrosine kinase receptor family, which is involved in cell proliferation. It has been identified as an oncodriver associated with breast, lung, gastric, colon, and other cancers development, and is related to worse prognosis [9-12]. Anti-HER2 therapy is well established in breast cancer, either in early-stage disease (as neoadjuvant or adjuvant therapy) or in an advanced stage; it is associated with an improved prognosis by increasing pathological complete response (pCR), disease-free survival (DFS), progression-free survival (PFS), and OS [13-17]. Many advances in anti-HER2 therapy have occurred such as the use of monoclonal antibodies (MoA), small molecule tyrosine kinase inhibitors (TKI), and antibody-drug conjugates (ADC). Trastuzumab, an MoA, was first approved for breast cancer patients, with proven activity in the metastatic setting as a single agent in the second line or beyond, and in the first line when combined with standard chemotherapy [15,18]. In the early setting, it has a benefit in DFS and OS as adjuvant treatment and provides an improved pCR as neoadjuvant therapy [19-21]. Some studies have demonstrated an improved benefit of trastuzumab when combined with other anti-HER2 therapy [13,16]. Trastuzumab therapy plays a role in other tumors treatment, like in gastric cancer as a first-line metastatic setting combined with chemotherapy [22]. ADC transformed HER2-targeted therapy by adding a chemotherapy component that will be delivered in a selected manner. Ado-trastuzumab emtansine (T-DM1) was first approved for breast cancer. More recently, trastuzumab deruxtecan (T-Dxd), an MoA conjugated with a topoisomerase I inhibitor, was approved. T-Dxd has demonstrated activity not only in HER2-positive breast cancer (overexpressed by immunohistochemistry (IHC, 3+) or amplified by in situ hybridization) but also in HER2-low tumors (defined as a score of 1+ on IHC analysis or as an IHC score of 2+ and negative results on in situ hybridization) [23]. In HER2-positive non-small cell lung cancer (NSCLC) and colorectal cancer, phase 2 studies have shown promising objective response rates - tumors without anti-HER2 therapy approved until now [24,25].

Data regarding HER2 expression and its clinical significance in HCC are contradictory. HER2 expression is reported in a range from 0% to 29.6% [26-30]. Some reports do not identify any association between HER2 expression and clinicopathological features, nor any relation to tumor development [27-29]. However, Shi et al., among others, recognized HER2 amplification as an early event in HCC tumorigenesis [31]. In this study, in the rat model, treatment with trastuzumab was associated with a decrease in the tumor size and in the advent of metastases, when compared with the control group [31]. Heinze et al. also identified an association between HER2 expression and poor prognosis, but this was not verified in other studies [28,32]. HER2 overexpression in HCC and its clinical significance need to be clarified as it can be a new targeted option in HCC.

This study aims to investigate HER2 expression in HCC and its clinical significance.

## Materials and methods

This was a single-center study conducted in a Portuguese hospital. Patients with histological diagnoses of HCC between 2010 and 2020 were included. Tissue specimens were analyzed by IHC. Clinicopathological features were accessed by medical records.

Tissue specimens

A total of 30 needle biopsies and 27 surgical specimens from a total of 54 patients were analyzed. In three patients, histological specimens from diagnosis and at recurrence were available.

Immunohistochemistry

For IHC staining, formalin-fixed paraffin-embedded tissues were cut into 3-5 µm thin sections. All reactions were performed using a Ventana Autostainer (Model Benchmark Ultra, Ventana Medical Systems, Roche, Tucson, AZ). Heat-induced epitope retrieval was performed in commercial pre-formulated CC1 (Ventana Medical Systems, Roche, Tucson, AZ) for 36 minutes at 95°C. Diagnostic antibodies against c-erbB-2 were analyzed (NeoMarkers, Fremont, CA; Clone 3B5, 1:1000 for 20 minutes). All reactions were developed using the Ventana UltraView DAB detection (Ventana Medical Systems, Roche, Tucson, AZ).

Scoring of HER2 staining

As there are no available guidelines for scoring HER2 expression in HCC, scoring criteria recommended by the College of American Pathologists for gastric cancer [22] were followed to determine the status of HER2 expression in these specimens. Thereby, HER2 membrane expression was evaluated.

For Surgical Specimens

Tumors without staining or with less than 10% of neoplastic cells with staining were scored as 0. Tumors with at least 10% of the cells with faint/barely perceptible staining were scored as 1+. Tumors with weak to moderate complete, basolateral, or lateral staining in at least 10% of the cells were considered equivocal and were scored as 2+. Those with at least 10% of the cells with strong and complete, basolateral or lateral membranous staining were considered to have overexpression and were scored as 3+.

For Biopsy Specimens

Tumors without staining were considered negative (score 0). Tumors with faint/barely perceptible staining in a cancer cell cluster (defined as a group of at least five neoplastic cells) were scored as 1+. Tumors with a cancer cell cluster with weak to moderate complete, basolateral, or lateral staining were considered equivocal and were scored as 2+. Those with a cancer cell cluster with strong and complete, basolateral or lateral membranous staining were considered to have overexpression and were scored as score 3+.

As there are no guidelines for HER2 staining classification in HCC, all the specimens with some HER2 reactivity were reported. An assessment of extra-membrane staining was performed and reported, but, when present, it was not considered an overexpression.

Statistical analyses

The association between tumor HER2 expression and BCLC staging, number of hepatic lesions, alpha-fetoprotein (AFP) level, underlying liver disease, presence of liver cirrhosis, and the Child-Pugh score was analyzed in all samples. The association of HER2 expression with tumor recurrence was analyzed in patients treated by surgery. The defined cutoff for AFP was 400 ng/mL.

DFS was analyzed in patients who had undergone surgery with curative intent (n = 27). OS analysis was performed in all samples. DFS was defined as the time from surgery to disease progression or death. OS was defined as the time from diagnosis to death from any cause.

Values were expressed as median and interquartile range. Mann-Whitney test was used to compare groups. Kaplan-Meier curves and log-rank tests were used for survival data. Test results were considered significant if p ≤ 0.05.

Ethical approval

Ethical approval for this study was provided by the Ethical Committee for Health, ULSM, Matosinhos, Portugal on May, 14th 2021 (Ethical Committee No.: 72/CES/JAS). Informed patient consent was waived because of the retrospective nature of the study.

## Results

Clinicopathological features

**Figure 1:**
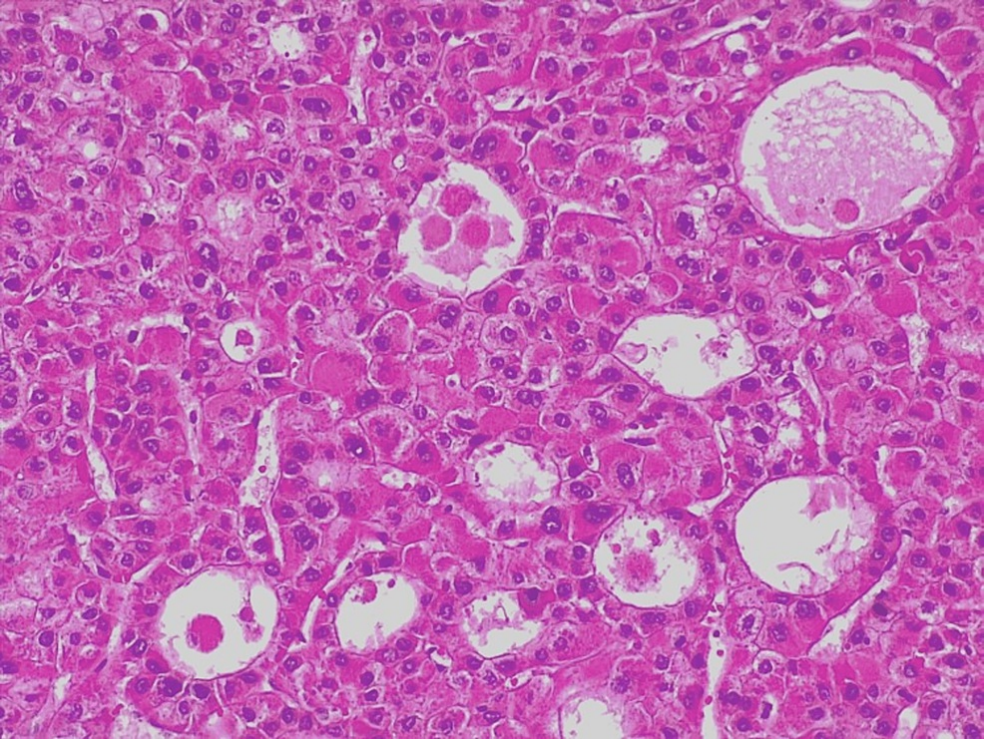
Hepatocellular carcinoma demonstrating pseudoacinar morphology with polygonal cells with atypical nuclei and eosinophilic cytoplasm. Hematoxylin and eosin staining (200x).

A total of 54 patients (46 men and eight women; median age: 71 years) with HCC (Figure 1) were included in this study. Clinicopathological features are summarized in Table 1. Of the patients, 57% had a solitary HCC nodule, with 61% at stage 0-A of the BCLC score. Alcohol-related liver disease was present in 44% of the patients, which was the most common cause of liver injury. Viral hepatitis was present in 37% (20% hepatitis C and 17% hepatitis B). Of the patients, 59% had cirrhotic liver. Regarding treatment, surgery was performed in 50%; 24% underwent local therapies (transarterial chemoembolization (TACE) or radiofrequency ablation (RAF)).

**Table 1:** Clinicopathological features of HCC patients. Columns three and four refer to patients with HER2 staining. * Eight patients have two etiologies for underlying liver disease (alcohol-related and viral hepatitis). + In cytoplasm staining, due to the small sample size, it is presented as minimum and maximum instead of IQR. AFP: alpha-fetoprotein; BCLC: Barcelona Clinic Liver Cancer; BSC: best supportive care; ECOG-PS: Eastern Cooperative Oncology Group performance status; HBV: hepatitis B virus; HCV: hepatitis C virus; HCC: hepatocellular carcinoma; HER2: human epidermal growth factor receptor 2; IQR: interquartile range; NASH: non-alcoholic steatohepatitis; RAF: radiofrequency ablation; TACE: transarterial chemoembolization.

	Study population		HER2 staining			
			Membrane		Cytoplasm	
	N = 54 (%)		N = 5 (%)		N = 2 (%)	
Age (years), median	71		70		66	
IQR^+^	59-75		63-76		59-73	
Male gender	46	(85)	4	(80)	1	(50)
ECOG-PS 0-1	48	(89)	4	(80)	2	(100)
AFP (ng/mL), median	11		4		45	
IQR^+^	2.4-130		1.8-28.6		2-87	
<400	45	(83)	5	(100)	2	(100)
Underling liver disease*						
Alcohol-related	24	(44)	3	(60)	2	(100)
NASH	3	(6)	1	(20)	-	-
HCV	11	(20)	-	-	-	-
HBV	9	(17)	1	(20)	-	-
Autoimmune	1	(2)	-	-	-	-
Non	3	(6)	-	-	-	-
Unknown	10	(19)	-	-	-	-
Liver cirrhosis						
Present	32	(59)	5	(100)	1	(50)
Unknown	5	(10)	-	-	-	-
Child-Pugh						
A	37	(69)	3	(60)	1	(50)
B	12	(22)	2	(40)	1	(50)
C	3	(6)	-	-	-	-
BCLC stage						
0-A	33	(61)	3	(60)	1	(50)
B	15	(27)	1	(20)	1	(50)
C	3	(6)	1	(20)	-	-
D	3	(6)	-	-	-	-
Hepatic lesions						
1	31	(57)	4	(80)	-	-
2-3	10	(19)	1	(20)	2	(100)
≥4	13	(24)	-	-	-	-
Treatment						
Surgery	27	(50)	2	(40)	2	(100)
TACE	8	(15)	1	(20)	-	-
RAF	5	(9)	1	(20)	-	-
Sorafenib	5	(9)	-	-	-	-
BSC	9	(17)	1	(20)	-	-

HER2 expression

HER2 staining was identified in the samples of seven patients (12%): four in biopsy specimens and three in surgical specimens. In five patients, there was membrane reactivity, and cytoplasm staining was noted in two patients.

**Figure 2:**
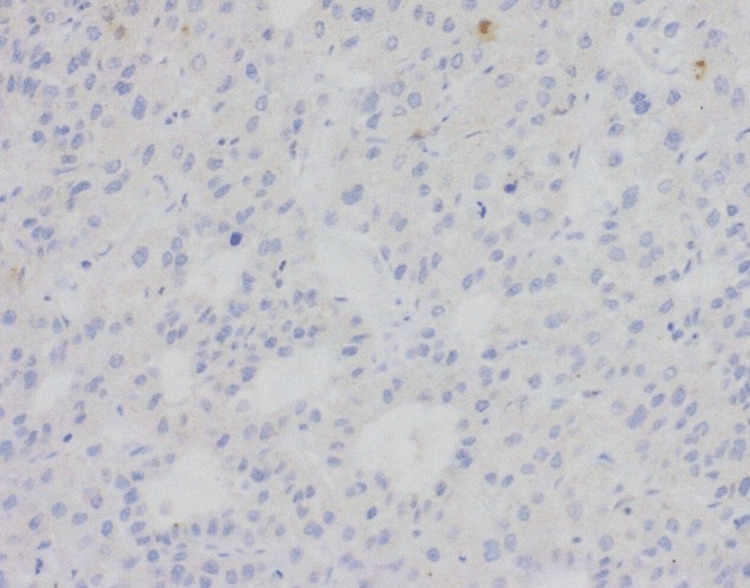
Surgical specimen without HER2 staining (score 0; 200x). HER2: human epidermal growth factor receptor 2.

**Figure 3:**
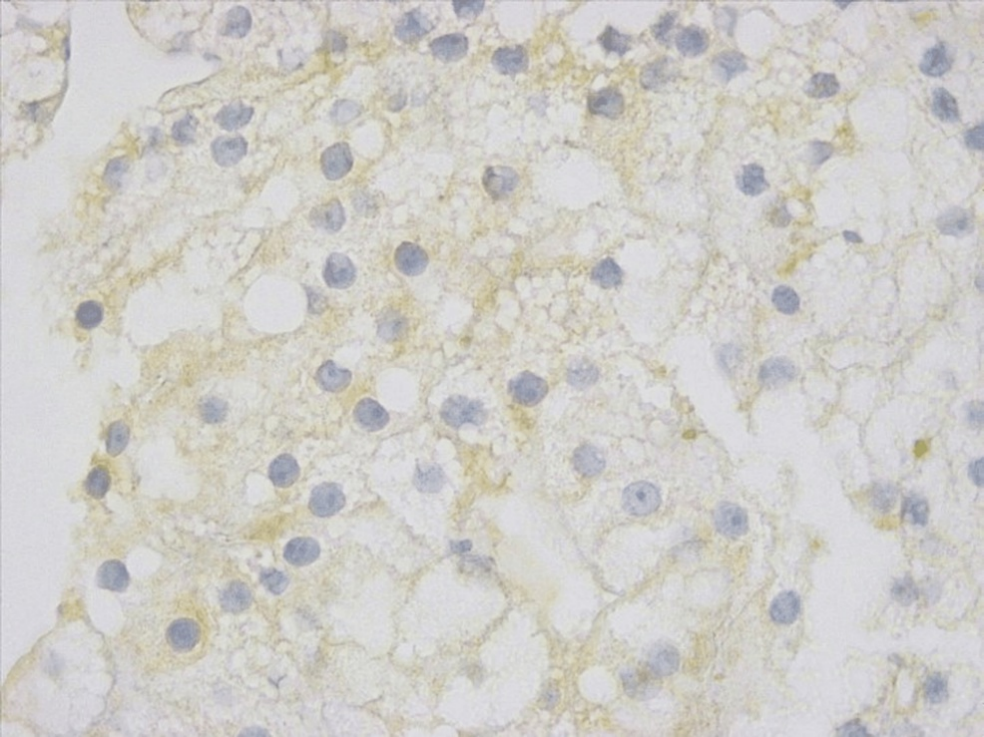
Biopsy specimen with a cancer cell cluster with faint/barely perceptible membranous reactivity (score 1+). Human epidermal growth factor receptor 2 (HER2) staining, 400x.

**Figure 4:**
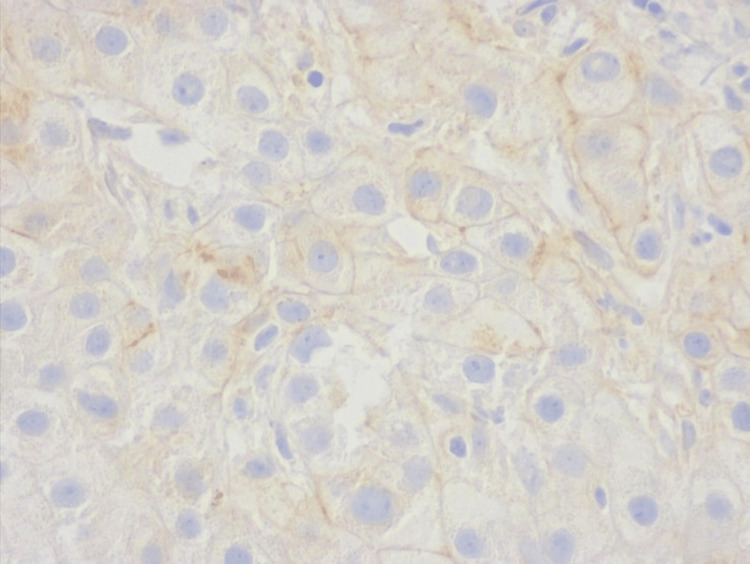
Biopsy specimens with a cancer cell cluster with weak to moderate complete and basolateral or lateral staining were considered equivocal (score 2+). Human epidermal growth factor receptor 2 (HER2) staining, 400x.

Regarding the membrane staining, none of the tumors had overexpression. One tumor presented a weak lateral membrane reactivity in 5% to 10% of tumor cells and was classified as score 0 (Figure 2). One patient had a biopsy specimen with a reactivity score classified as 1+ (Figure 3). In three patients, the staining was classified as score 2+ (Figure 4). In these patients, the staining was weak and on the lateral membrane in all cases. So, an IHC score of 1+/2+ was identified in 7.4% (n = 4) of the patients.

**Figure 5:**
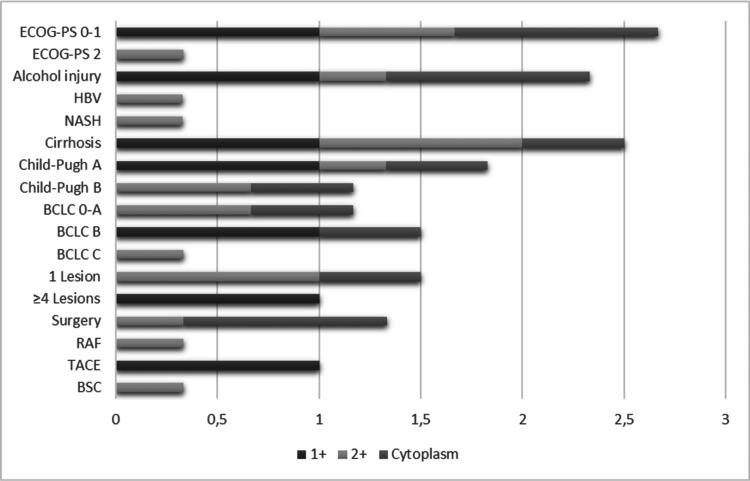
Proportion of HER2 staining by clinicopathologic characteristics. In patients with HER2 2+ score, HCC presented as a single node in all the cases. The BCLC C score was due to an ECOG-PS of 2. Regarding underlying liver disease, it was alcohol-related in one patient, NASH in another, and HBV in the other of the HER2 2+ score. Patients with a 1+ score and cytoplasm staining liver injury were alcohol-related in all cases. BCLC: Barcelona Clinic Liver Cancer; BSC: best supportive care; ECOG-PS: Eastern Cooperative Oncology Group performance status; HBV: hepatitis B virus; HCC: hepatocellular carcinoma; HER2: human epidermal growth factor receptor 2; NASH: non-alcoholic steatohepatitis; RAF: radiofrequency ablation; TACE: transarterial chemoembolization.

Regarding clinicopathological features, detailed in Table 1 and Figure 5, the disease was at BCLC stage 0-A and there was a single HCC lesion in four patients. AFP was <400 ng/mL in all patients who presented some HER2 reactivity. Liver injury was alcohol-related in five patients, one had hepatitis B and the other had non-alcoholic steatohepatitis (NASH).

All patients were treated with surgery or local therapies except one. That patient was proposed to best supportive care due to comorbidities.

Of the patients who underwent surgery, 75% (n = 3) had tumor recurrence. Only one patient with HER2 expression had histological material available at tumor progression. In this case, the staining was scored as 2+ at the diagnosis and had disappeared with tumor progression. No clinicopathological differences between patients with HER2 scores 1+/2+ and score 0 were identified.

Survival data

Median follow-up was 20 months (1-140), and 42 events (progression or death of any cause) occurred during this period. DFS was analyzed in surgically resected HCC (n = 27). Median DFS was 26 months (7-44). When evaluated by HER2 expression, the median DFS in HER2 score 1+/2+ and cytoplasmatic was 38 months versus 22 months in HER2 non-expression patients, without statistical significance (p = 0.604). Median OS was 29 months (18-40) in all populations.

## Discussion

HCC maintains a poor prognosis despite efforts to understand molecular pathogenesis and to identify targeted mutations [1,33]. The genetic analysis allowed the identification of the main drivers responsible for tumor initiation and progression, such as telomere reverse transcriptase (TERT) and fibroblast growth factor 19 (FGF19) mutations, inactivation of tumor antigen p53 (TP53), and activation of Wnt, mammalian target of rapamycin (mTOR), and RAS signaling pathways [33]. A recent analysis of genes expressed in normal liver samples and HCC samples identified HER2 as one of the top 10 key genes in tumor initiation and progression [34]. Shi et al. corroborated this finding by recognizing HER2 amplification as an early event in HCC tumorigenesis that declines with increasing tumor grade and stage [31]. Döring et al., in a study of nuclear HER2 expression in hepatocytes in liver disease, identified HER2 positivity as a recent event in HCC development, but that unlikely is conserved in the progression to preneoplastic lesions [35]. Some studies had investigated HER2 amplification/overexpression in HCC, and the data are contradictory. Vlasoff et al. and Panvichian et al. did not identify HER2 expression in HCC [26,36]. By contrast, in other studies, HER2 was overexpressed in a range of 2.1% to 29.6% of tumor cells, but no association with the malignant phenotype was established [27-29]. In this study population, we identified HER2 expression in seven patients (12%). In two of them, it was a cytoplasmatic staining and one of the membrane staining was classified as an IHC score of 0. So, countable staining was present in only 7.4% of the patients and scored as 1+ or 2+. These results are in accordance with historical studies suggesting that HER2 expression is a rare event in HCC. Nevertheless, similar to Shi et al.'s findings, the HER2 expression seems to be an early event that disappears with tumor progression. This is justified by HER2 expression disappearance with tumor progression, associated with all tumors scored as 2+ by IHC presenting as a single HCC lesion. However, we cannot draw these conclusions because only one HER2 staining patient had specimens at diagnosis and recurrence, and no association between the number of nodules and HER2 expression was found.

In this sample, it was not possible to establish a relation between HER2 expression and clinicopathologic features. However, regarding the underlying liver disease, in patients with an IHC score of 1+ or 2+, the disease was alcohol-related in two patients, with hepatitis B and NASH in the other two. All of these cases had surrounded liver cirrhosis. In Döring et al.'s study on non-cancerous liver disease, a strong association was identified between HER2-positive cases and alcoholic steatohepatitis (ASH), which was not verified in NASH. Scores of hepatocellular HER2 expression were significantly correlated with ASH and NASH inflammatory activity. By contrast, in viral and autoimmune hepatitis, HER2 immunoreactivity was negative in most cases, and independent of inflammatory activity [35]. Brunt et al. identified HER2 as an epiphenomenon of hepatitis B or hepatitis C virus infection [37]. Otherwise, Hung et al. provided evidence that hepatitis B virus (HBV)-encoded X (HBx) protein, a regulatory protein that contributes to HCC tumor progression, upregulates HER2 expression through HuR-dependent mRNA stabilization [38]. It also identified a more proportion of metastatic cells [38]. However, other studies like ours had not found a relationship between clinicopathologic features and HER2 expression [27-29].

Regarding how HER2 expression influences the prognosis, the data remain contradictory. In our sample, HER2 expression was associated with an improvement in DFS, without statistical significance. This result was contrary to what we were expecting once HER2 expression was generally associated with a poor prognosis in other tumors [12,39]. In HCC, Heinze et al. identified an association between HER2 expression and poor prognosis, but in studies by Nakopoulou et al. and Ito et al., this was not verified [27,28,32]. So, this still needs clarification.

A study demonstrated that the pattern of hepatocellular HER2 expression differs from breast cancer cells [35]. Staining was most frequently strongly nuclear and more or less intensely cytoplasmic; rarely, an exclusive membrane expression was observed. A strong complete membrane expression, like score 3+ in breast cancer, was never observed in hepatocytes of liver disease [35]. In another study, HER2 staining was reported mostly as faint or weak, having only one patient with strong membranous staining [29]. The same was identified in this study, where HER2 staining was either faint or weak at the membrane or cytoplasmatic. Furthermore, once HER2 has been identified as one of the main genes involved in tumor initiation and progression, it will be interesting to think about other mechanisms of HER2 activation, such as HER2 mutation, as occurs in NSCLC [11,34]. In NSCLC, HER2 activating mechanisms can be gene mutation, gene amplification, and protein overexpression, and the gene mutation is not associated with overexpression [11]. This reinforces the need to create proper guidelines for classifying HER2 expression in HCC since these are not transversal to all tumor types [22,40].

Regarding anti-HER2 therapy in HCC, data are limited due to its low expression. Shi et al. demonstrated that treatment with trastuzumab 21 days after HCC cell inoculation and major hepatectomy (that had previously been associated with rapid cancer growth) in rats’ models was associated with a significant decrease in the tumor size and metastases [31]. In the same study, in the in vitro analysis, trastuzumab did not confer with a survival decrease in tumor cells. However, when cell proliferation ability was assessed through epidermal growth factor stimulation in a serum-free medium, trastuzumab inhibited cell proliferation [31]. Hsu et al. did not identify any inhibitory activity of trastuzumab in vitro HCC cell growth [30]. In other tumor types, the antitumor activity of HER2-directed therapy is well established. In breast cancer patients, the addition of trastuzumab to standard chemotherapy in the first-line setting of irresectable or metastatic cancer provides a 20% reduction in the risk of death [15]. This benefit increase by 32% when pertuzumab is added to the chemotherapy-trastuzumab regimen [16]. The benefit of maintaining anti-HER2 therapy after progression on HER2-directed therapy is evident, either by changing to another type of anti-HER2 therapy (ADC or TKI) or, in advanced lines, by trastuzumab reintroduction [18,41-43]. In the early setting, as adjuvant therapy, one year of trastuzumab reduces the mortality risk by 37% and improves DFS by 40% [19,20]. When in neoadjuvant therapy, it is associated with an improved pCR, achieving an additional response when the dual blockade is used - rates of 46% pCR [13,21]. It is also known that patients who achieve a pCR had better outcomes, namely, an improved OS [13]. T-Dxd had some positive results in many studies. In the phase II DESTINY-Breast01 trial, in heavily pretreated patients, the overall response rate (ORR) was 62%, and the median OS was 29.1 months [44]. In the phase III DESTINY-Breast03 trial, as a metastatic second-line, T-Dxd provided a reduction in 45% of the risk of death when compared to T-DM1, and an ORR of 79.7% versus 34.2%, respectively [45]. This benefit was extended even to HER2 low patients, as demonstrated in DESTINY-Breast04, with a hazard ratio for death of 0.64 in all populations [23]. Also, in other tumor types, HER2-directed therapy is effective; trastuzumab with fluoropyrimidine-based chemotherapy is the standard metastatic first-line in HER2-positive gastric cancer [22]. In NSCLC, poziotinib, a TKI, had demonstrated activity in 12 heavily pre-treated patients with HER2 exon-20 insertion, as it was T-DM1 (ORR of 42% and 38.1%, respectively) [46,47]. The great benefit in these patients was observed with T-Dxd with an ORR of 61% and a PFS of 14 months [25]. T-Dxd also has proven efficacy in colorectal cancer (ORR: 45.3%) [24]. It is clear that HER2 therapy has emerged in cancer treatment and is still evolving, improving the outcomes of different tumors. HCC treatment is still meager. At a very early stage, surgery is the treatment of choice, having a curative intent, despite that recurrence rates are high (50-70%) [1]. When surgery is not possible, and patients meet Milan criteria, transplantation is offered. However, even with a judicious selection of patients for liver transplantation, tumor recurrence occurs in 15-20% [3]. Regarding local therapies, RAF is the most effective, but it has local tumor progression rates and distant recurrence at five years of 3.7-27% and 74.8%, respectively [48,49]. To improve these outcomes, some studies have explored the possibility of systemic adjuvant therapy. The STROM trial, which assessed sorafenib versus placebo as adjuvant therapy in patients who had undergone surgery or ablation, failed to achieve its primary end-point: recurrence-free survival (RFS) and median RFS was 33.3 and 33.7 months, respectively [50]. In HBV-related HCC, interferon α as adjuvant therapy had prolonged RFS in two studies when compared to placebo [51,52]. In a meta-analysis of 14 studies, interferon α improves OS and RFS in hepatitis C virus (HCV)-related HCC, and the OS but not the RFS in HBV-related HCC [53]. So, the treatment of viral hepatitis is widely used to prevent new tumor lesions. Immune checkpoint inhibitors (ICI) had proven efficacy in advanced HCC, being the standard first-line based on IMbrave150 [7]. It also has proven benefits as an adjuvant treatment in other tumors like esophagus and melanoma [54,55]. Some clinical trials were designed to assess the use of ICI in the adjuvant setting. NIVOLE, a phase II trial, evaluated the efficacy of nivolumab as an adjuvant treatment: the RFS at one year was 78.6%, and the median RFS was 26.3 months [56]. Many clinical trials with ICI +/- bevacizumab in this adjuvant setting are in progress (CheckMate 9DX; KEYNOTE-937; EMERALD-2; JUPITER 04; IMbrave050). TACE was also evaluated as an adjuvant treatment after surgery; it had an improved RFS and OS (HR: 0.7) compared with the placebo, in two clinical trials [57,58]. Despite the efforts to improve early HCC outcomes, no adjuvant treatment had the impact to change the clinical practices and no regimen is approved or recommended by international guidelines [59,60]. The advances in HER2-directed therapy, namely, targetable HER2 low tumors, combined with Shi et al.'s findings [31], make it reasonable to think about adjuvant HER2-directed therapy in very early HCC patients.

This study has some limitations. It is a unique center retrospective study, and the sample size is small. Due to its retrospective nature, there are few patients with specimens from the diagnosis and the recurrence, which limits the conclusion we can draw on the behavior of HER2 expression with tumor progression. Additionally, HCC is mostly diagnosed by pathognomonic features on imaging tests, which limits the number of available samples and produces a selection bias.

## Conclusions

In this study, HER2 expression was a rare event (7.4%), and no relation between clinicopathological features and HER2 expression was established. Despite that, and as HER2-targeted therapy is evolving, it is important to continue this investigation and draw guidelines for HER2 expression classification in HCC. In addition to the evaluation of HER2 expression, other markers have to be explored to improve the treatment of HCC.
